# Structure Revision and Protein Tyrosine Phosphatase Inhibitory Activity of Drazepinone

**DOI:** 10.3390/md19120714

**Published:** 2021-12-20

**Authors:** Fei Cao, Li Pan, Wenbin Gao, Yunfeng Liu, Caijuan Zheng, Yahui Zhang

**Affiliations:** 1 Key Laboratory of Pharmaceutical Quality Control of Hebei Province, Key Laboratory of Medicinal Chemistry and Molecular Diagnostics of Education Ministry of China, College of Pharmaceutical Sciences, Hebei University, Baoding 071002, China; liuyunfeng199011@163.com; 2 State Key Laboratory of NBC Protection for Civilian, Beijing 102205, China; bk6180b@163.com; 3 College of Life Sciences, Cangzhou Normal University, Cangzhou 061000, China; wenbinxing@yeah.net; 4 Key Laboratory of Tropical Medicinal Resource Chemistry of Ministry of Education, Hainan Normal University, Haikou 571158, China; 5 Key Laboratory of Marine Drugs, The Ministry of Education of China, School of Medicine and Pharmacy, Ocean University of China, Qingdao 266003, China

**Keywords:** *Penicillium sumatrense* (Trichocomaceae), structure revision, drazepinone, PTP inhibitory activity, molecular docking

## Abstract

From the marine-derived fungus *Penicillium sumatrense* (Trichocomaceae), a pair of enantiomers [(+)-**1** and (−)-**1**] were isolated with identical 1D NMR data to drazepinone, which was originally reported to have a trisubstituted naphthofuroazepinone skeleton. In this study, we confirmed the structures of the two enantiomers as drazepinone and revised their structures by detailed analysis of extensive 2D NMR data and a comparison of the calculated ^13^C chemical shifts, ECD, VCD, and ORD spectra with those of the experiment ones. (+)-**1** and (−)-**1** were evaluated for their PTP inhibitory activity in vitro. (−)-**1** showed selective PTP inhibitory activity against PTP1B and TCPTP with IC_50_ values of 1.56 and 12.5 μg/mL, respectively.

## 1. Introduction

**Figure 1:**
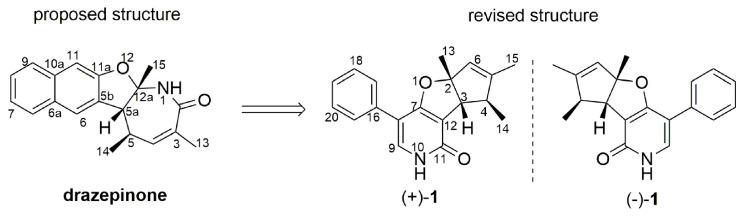
The proposed and revised structures of drazepinone.

Without the assistance of single-crystal X-ray diffraction analysis, many complex natural products that exhibited important biological activities and played a major role in drug discovery were assigned to erroneous structures or could not be unambiguously established [1]. Drazepinone (a previously proposed structure as a trisubstituted tetrahydronaphthofuroazepinone core; Figure 1), a specialized metabolite that is a type of alkaloid, was obtained for the first time in 2005 from a pathogenic fungal strain of *Drechslera siccans* from seeds of *Lolium perenne* [2]. In biological activity studies, drazepinone has been proved to have broad-spectrum herbicidal properties with low zootoxic activity at a concentration of 2.0 μg/μL, suggesting that it could be applied as an environmentally friendly and safe herbicide [2]. During the course of our ongoing research on bioactive natural products from marine-derived fungi [3,4], an extract of the fungal strain *Penicillium sumatrense* (Trichocomaceae) showed inhibitory activity against protein tyrosine phosphatase (PTP). Our chemical investigations of this fungus using a bioassay-guided method led to the isolation of compounds (+)-**1** and (−)-**1**, comprising a racemate with identical 1D NMR data to drazepinone. However, we found that some 2D NMR data of drazepinone (^1^H-^1^H COSY and key HMBC) were incorrectly interpreted, prompting us to carefully analyze the NMR spectroscopic data of (+)-**1** and (−)-**1**, and to revise the structure of drazepinone (Figure 1).

By the rapid development of modern strategies and methods for structural elucidation, DFT calculations of ^13^C chemical shifts and chiral spectra proved to be critical in several profile structure revisions and have greatly facilitated the reliable determination of natural products with undescribed structures [5,6]. In the present work, we thus tried to definitively determine the structure of drazepinone by detailed analysis of 2D NMR data and quantum-mechanics-based computational studies of ^13^C chemical shifts, electronic circular dichroism (ECD), vibrational circular dichroism (VCD), and optical rotatory dispersion (ORD) of (+)-**1** and (−)-**1**. Here we report a definitive revision of the structures of (+)-**1** and (−)-**1** and also describe their PTP inhibitory activity.

## 2. Results and Discussion

### 2.1. Structural Elucidation and Revision

The fungal strain *Penicillium sumatrense* was cultivated using a rice medium. Purification of the EtOAc extract of the fungus by HPLC yielded **1**. It is worth noting that the optical rotation of **1** was near zero, indicating its racemic nature. Finally, the mixture of (±)-**1** was separated using a Chiralpak IB column to yield (+)-**1** and (−)-**1**.

**Figure 2:**
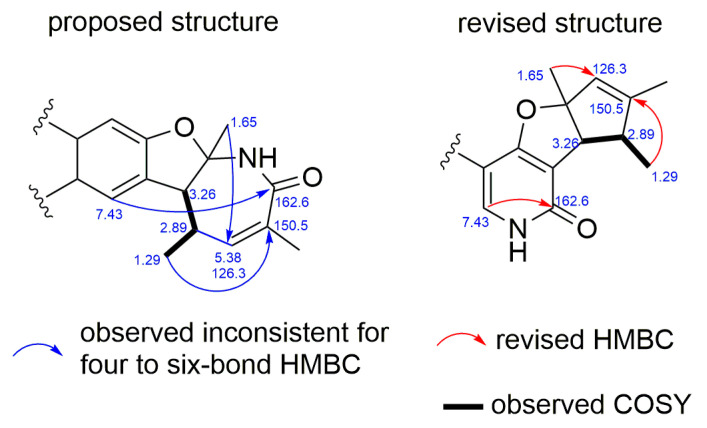
Observed inconsistent and revised 2D NMR data of drazepinone.

**Figure 3:**
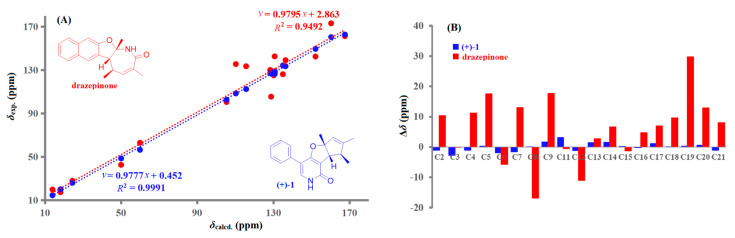
Regression analysis (**A**) and individual deviations (**B**) of experimental versus calculated ^13^C NMR chemical shifts of drazepinone and (+)-**1**.

Compounds (+)-**1** and (−)-**1** were isolated as white amorphous substances with the molecular formula C_19_H_19_NO_2_ on the basis of their HRESIMS data at *m*/*z* 294.1464 [M + H]+ ion. The ^1^H and ^13^C NMR data of (+)-**1** and (−)-**1** (Table 1 and Table S1) displayed three methyls, nine methines including seven aromatic carbons, and seven non-protonated carbons. These findings were in agreement with 1D NMR and MS data reported for drazepinone [2]. However, in the interpretation of the ^1^H-^1^H COSY and key HMBC data for (+)-**1** and (−)-**1**, some important correlations were inconsistent with the proposed structure of drazepinone. For example, when the HMBC correlations for the proposed structure were interpreted, strong HMBC correlation from H-6 (*δ*_H_ 7.43) to C-2 (*δ*_C_ 162.6), an unreasonable six-bond correlation, could not be well explained (Figure 2). Similarly, the HMBC correlations from H-14 (*δ*_H_ 1.29) to C-3 (*δ*_C_ 150.5), and from H-15 (*δ*_H_ 1.65) to C-4 (*δ*_C_ 126.3), which represent uncommon four-bond and five-bond correlations, respectively, were not taken into consideration for the proposed structure of drazepinone. To further confirm the above deduction, ^13^C NMR chemical shift calculation based on the gauge-independent atomic orbital (GIAO) [7] was performed for the proposed structure of drazepinone at the level of B3LYP/6-311G+(d,p). Unfortunately, a very low correlation coefficient (*R*^2^) of 0.9492 was given for the proposed structure (Figure 3). Moreover, this procedure gave a mean deviation (|∆*δ*|_mean_) of 6.1 ppm and a maximum deviation (|∆*δ*|_max_) of 29.9 ppm between the experimental and calculated (corrected) ^13^C chemical shifts for the wrong structural assignment (Figure 3). Thus, the structures of (+)-**1** and (−)-**1** should be reassigned.

**Figure 4:**
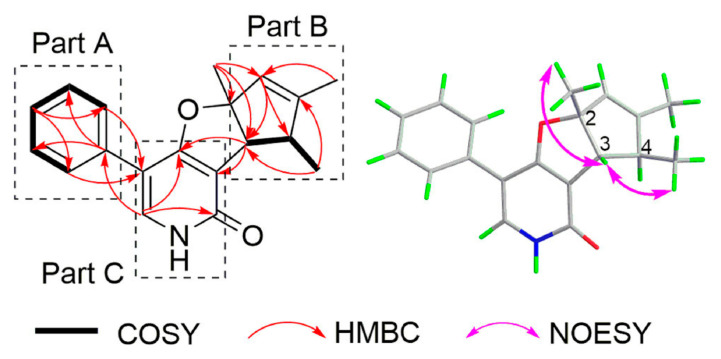
COSY, key HMBC, and key NOESY correlations of (+)-**1** and (−)-**1**.

According to the 1D and 2D NMR data (Figures S1–S13), it could be deduced that (±)-**1** consisted of three units, phenyl group (Part A), trimethylcyclopent (Part B), and furopyridone (Part C) (Figure 4). The key HMBC correlations from H-17/19 to C-8, and from H-9 to C-16 suggested the C-C bond between C-8 and C-16. Moreover, the connectivity between oarts B and C was established by the key HMBC correlations from H-3 to C-7 and C-12. Thus, the planar structures of (+)-**1** and (−)-**1** were established, with a 6-6/5/5 ring system. Importantly, in the revised structures of (+)-**1** and (−)-**1**, the HMBC correlations from *δ*_H_ 7.43 to *δ*_C_ 162.6, from *δ*_H_ 1.29 to *δ*_C_ 150.5, and from *δ*_H_ 1.65 to *δ*_C_ 126.3, all of which were three-bond correlations, and the absence of COSY correlation between *δ*_H_ 2.89 and *δ*_H_ 5.53, could be taken into consideration perfectly (Figure 2). Furthermore, GIAO ^13^C NMR calculation was also performed for the revised structure (+)-**1** at the same level as that for the proposed structure of drazepinone. As shown in Figure 3, the max individual deviation (|Δ*δ*|) between the experimental and calculated chemical shifts was 3.1 ppm for (+)-**1**. Additionally, the high correlation coefficient (*R*^2^ = 0.9991) for (+)-**1** (Figure 3), indicating that the *δ*_C_ of (+)-**1** matched the calculated *δ*_C_ very well, which confirmed the framework of the revised structure of (+)-**1**. Detailed analysis of ^13^C NMR data of (+)-**1** and (−)-**1**, it was strangely found that the chemical shift of C-2 with value of *δ*_C_ 102.2 (DMSO-*d*_6_)/105.3 (CD_3_OD)/103.9 (CDCl_3_) were much lower than what was expected, and the ^13^C NMR chemical shift calculation of (+)-**1** showed that this phenomenon was entirely possible, which was caused by a combination of oxygen and double bond. Subsequently, NOESY correlations from H-3 (*δ*_H_ 3.08) to H_3_-13 (*δ*_H_ 1.58) and H_3_-14 (*δ*_H_ 1.17) (Figure S13) indicated that all of these protons should be placed on the same side of the molecule (Figure 4).

**Figure 5:**
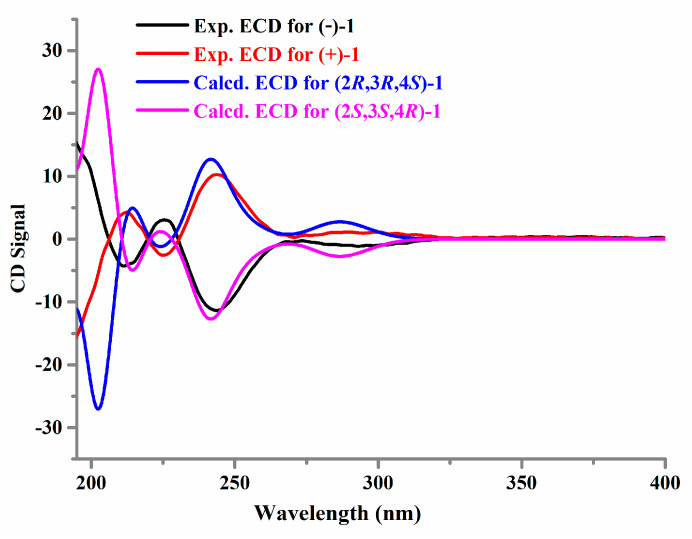
Experimental and calculated ECD spectra of (+)-**1** and (−)-**1**.

Determination of the absolute configurations by the ECD method combined with quantum-mechanical calculations [8,9] was carried out for (+)-**1** and (−)-**1**. Based on the relative configuration of (+)-**1** and (−)-**1**, molecules (2*R*,3*R*,4*S*)-**1** and (2*S*,3*S*,4*R*)-**1** were chosen for conformational searches, structural optimizations, and time-dependent density functional theory (TD-DFT) ECD calculations. As shown in Figure 5, the experimental ECD spectra of (+)-**1** and (−)-**1** had good agreement with the calculated ECD spectra of (2*R*,3*R*,4*S*)-**1** and (2*S*,3*S*,4*R*)-**1**, respectively. Thus, the absolute configurations of (+)-**1** and (−)-**1** were assigned as 2*R*,3*R*,4*S* and 2*S*,3*S*,4*R*, respectively. Furthermore, the proposed structure of drazepinone was also used for TD-DFT ECD calculation. The calculated results showed that neither the experimental ECD spectrum of (+)-**1** nor (−)-**1** matched well with the calculated ECD spectrum of the proposed structure of drazepinone (Figure S15), confirming our structural revision.

**Figure 6:**
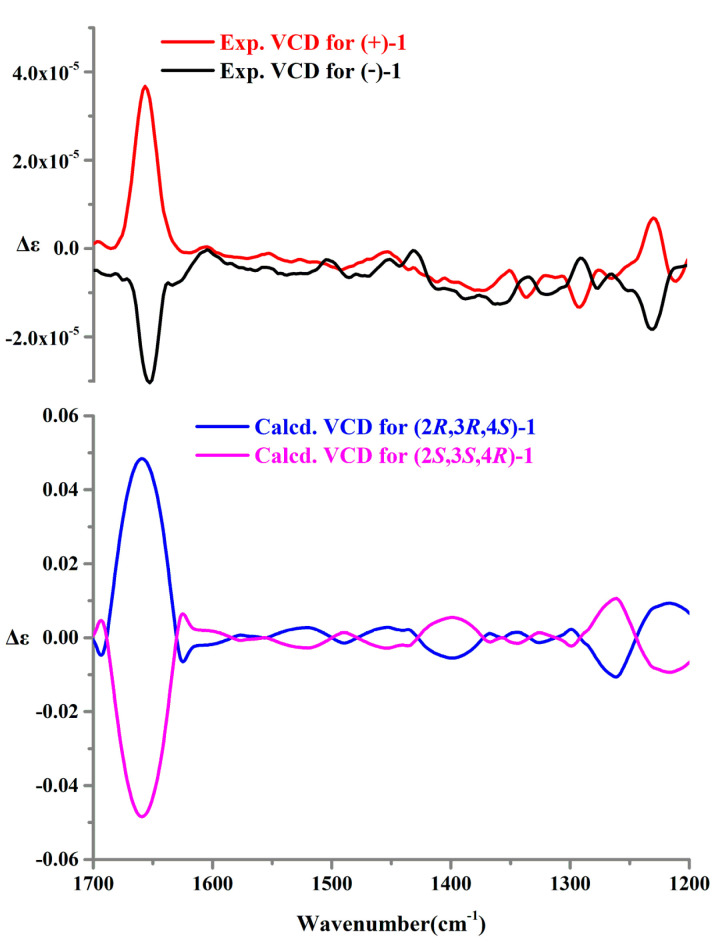
Experimental and calculated VCD spectra of (+)-**1** and (−)-**1**.

**Figure 7:**
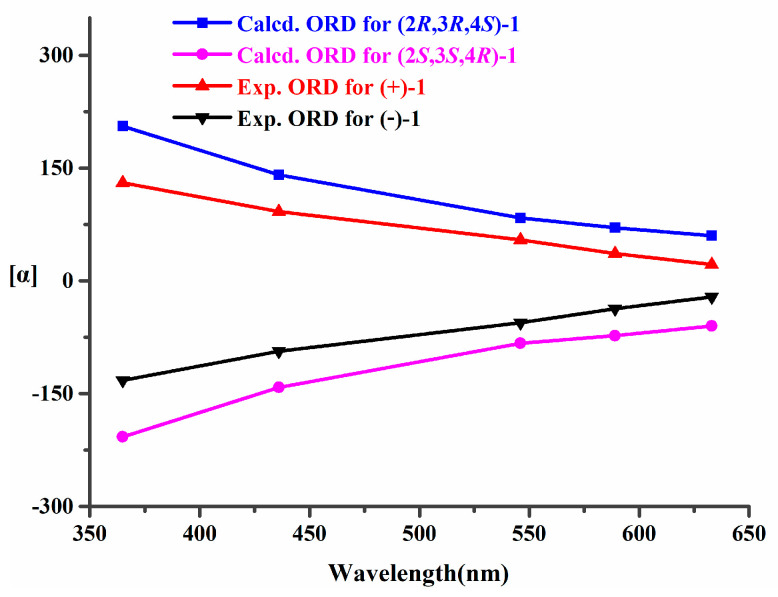
Experimental and calculated ORD data of (+)-**1** and (−)-**1**.

Recently, in addition to ECD, other chiroptical techniques, such as VCD and ORD, have also been commonly employed to assign the absolute configurations of some previously undescribed natural products, especially using a combined analysis of ECD, VCD, and ORD properties [10,11,12,13]. Thus, we tried to further confirm the absolute configurations of (+)-**1** and (−)-**1** by using VCD and ORD. Firstly, the experimental VCD spectra in DMSO-*d*_6_ of (+)-**1** and (−)-**1** were carried out at a resolution of 4 cm^−1^, and the experimental ORD data for (+)-**1** and (−)-**1** at five wavelengths (365, 436, 546, 589, and 633 nm) were recorded in CH_3_OH. Then, the calculation of VCD and ORD spectra for (2*R*,3*R*,4*S*)-**1** and (2*S*,3*S*,4*R*)-**1** were carried out. Finally, the experimental VCD and ORD data of (+)-**1** and (−)-**1** were combined for analysis with their corresponding theoretical predictions (Figure 6 and Figure 7). The results verified that the absolute configurations of (+)-**1** and (−)-**1** were unambiguously assigned as 2*R*,3*R*,4*S* and 2*S*,3*S*,4*R*, respectively.

**Scheme 1:**
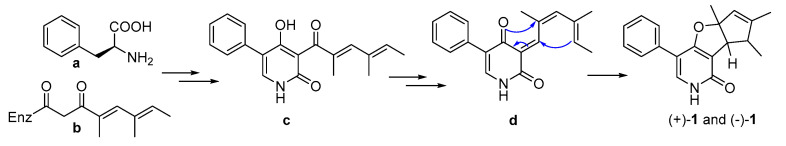
Hypothetical biosynthetic pathways for (+)-**1** and (−)-**1**.

A plausible biogenetic pathway of (+)-**1** and (−)-**1** is proposed as shown in Scheme 1. The important presumed intermediate b is synthesized via the acetyl malonyl pathway. Intermediate c, which is derived from a and b, produced the intermediate d. Then, through cycloisomerization reaction, (+)-**1** and (−)-**1** were obtained from d.

### 2.2. PTP Inhibitory Activity

Protein tyrosine phosphatases (PTPs), including PTP1B, T-cell PTP (TCPTP), Src homology-2 domain-containing PTP-1 (SHP1), and CD45 PTP (CD45), a family of enzymes that hydrolytically remove phosphate groups from proteins, have attracted much attention recently for drug discovery [14,15,16]. Among which, several alkaloids have been reported to exhibit potent inhibitory activity against PTPs [15,16]. Thus, the isolated compounds (+)-**1** and (−)-**1** were tested for their PTP inhibitory activity against PTP1B, TCPTP, SHP1, and CD45. The bioassay results (Table 2) informed that (−)-**1** showed selective inhibitory activity against PTP1B and TCPTP, with the IC_50_ values of 1.56 and 12.5 μg/mL, respectively. However, (+)-**1** exhibited no activity against any of the PTPs, suggesting that the configurations of chiral carbons in (+)-**1** and (−)-**1** may play an important role in PTP inhibitory activity.

**Figure 8:**
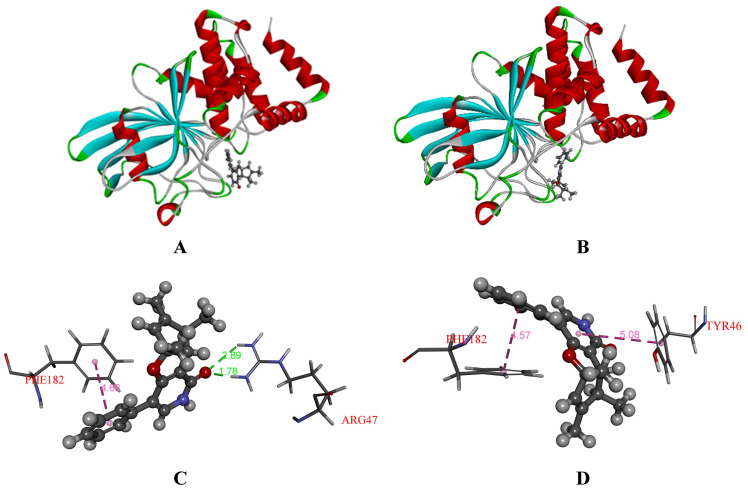
(**A**) Docked ligands [(−)-**1**] in the binding pocket of PTP1B (PDB: 5T19); (**B**) docked ligands [(+)-**1**] in the binding pocket of PTP1B (PDB: 5T19); (**C**) interactions between (−)-**1** and PTP1B (PDB: 5T19); (**D**) interactions between (+)-**1** and PTP1B (PDB: 5T19). The H-bonds were shown in green. The π-π interaction were shown in pink.

**Figure 9:**
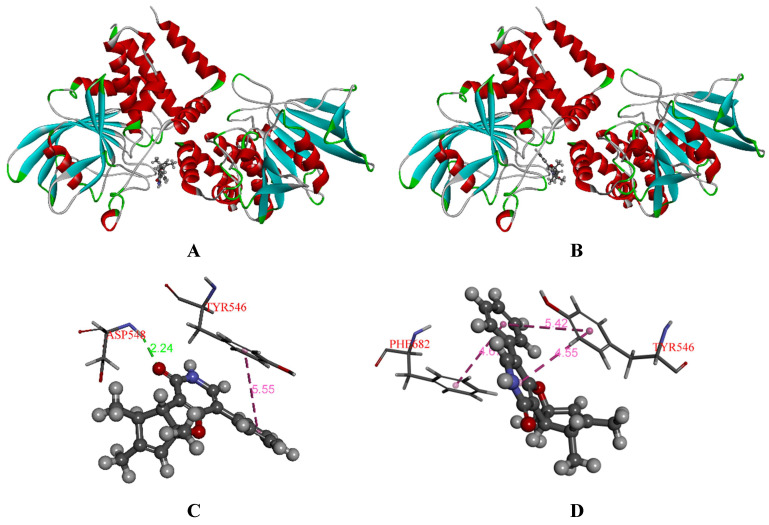
(**A**) Docked ligands [(−)-**1**] in the binding pocket of TCPTP (PDB: 2FJM); (**B**) docked ligands [(+)-**1**] in the binding pocket of TCPTP (PDB: 2FJM); (**C**) interactions between (−)-**1** and TCPTP (PDB: 2FJM); (**D**) interactions between (+)-**1** and TCPTP (PDB: 2FJM). The H-bonds were shown in green. The π-π interaction were shown in pink.

To further explore the difference in inhibitory effects between (+)-**1** and (−)-**1** against PTP1B and TCPTP, the molecules (2*R*,3*R*,4*S*)-**1** and (2*S*,3*S*,4*R*)-**1** were selected to investigate the binding mode using molecular docking. The conformations of ligands in the binding pocket of PTP1B and TCPTP were shown in Figure 8 and Figure 9. The docked conformation of (−)-**1** was found to tightly bind to the entire active pocket of PTP1B or TCPTP. Docking results implied that (−)-**1** binds deep in the active site pocket and forms H-bonds with the ARG47 of PTP1B (Figure 8C), while, interestingly, there was no H-bond to stabilize (+)-**1** in PTP1B (Figure 8D). Similarly, H-bonds were found between (−)-**1** and residue ASP548 in TCPTP (Figure 9C), but not for (+)-**1** in TCPTP (Figure 9D). Therefore, H-bonds could make the interaction between (−)-**1** and PTP1B/TCPTP stronger than that between (+)-**1** and PTP1B/TCPTP.

## 3. Materials and Methods

### 3.1. General Experimental Procedures

ORD data were measured using a JASCO P-2000 spectrometer in CH_3_OH. UV and ECD spectra were performed using a Perkin-Elmer model 241 spectrophotometer and a JASCO J-715 CD spectrometer, respectively. VCD spectra, including the corresponding IR spectra, were acquired using a BioTools Chiral*IR*-2X spectrophotometer. NMR data with TMS as an internal standard were measured using Bruker Avance-Ⅲ 600 MHz NMR spectrometer. HRESIMS data were obtained from a Bruker apex-ultra 7.0T spectrometer. Semipreparative HPLC, using Waters (XBridge OBD, 5 μm, 10 × 250 mm) and Daicel (Chiralpak IB, 5 μm, 10 × 250 mm) columns, was carried out on a Shimadzu LC-20AT system with a SPD-M20A photodiode array detector. Column chromatography was performed on Silica gel 200–300 and 300–400 mesh, and Sephadex LH-20 18−110 μm.

### 3.2. Isolation of Fungal Material

The marine-derived fungus *Penicillium sumatrense* (Trichocomaceae; GenBank accession number MZ779032) was collected from the Bohai Sea in June 2018. Rice fermentation of *P. sumatrense* was carried out using 60 Erlenmeyer flasks (1000 mL, containing 100 g rice and 100 mL distilled H_2_O). The fungal material, fermented at 28 °C for 45 days, were extracted with methanol for three times, then further concentrated in vacuo and extracted using EtOAc two times to give a dark oily residue (47.0 g). The extract was subjected to silica gel column chromatography (CC) with a gradient elution of petroleum ether (PE)/EtOAc (90:10, 50:50, 10:90 (*v*/*v*)) to give three fractions, Frs. 1–3. Fr. 2, which was repeatedly purified by silica gel CC, ODS silica gel, and Sephadex LH-20, was then purified by semipreparative HPLC using an XBridge column (60% MeOH/H_2_O, 2.0 mL/min) to obtain **1** (6.0 mg). Finally, the mixture of (±)-**1** was separated using a Chiralpak IB column (70% PE/EtOH, 2.0 mL/min) to yield (+)-**1** (2.8 mg) and (−)-**1** (2.9 mg).

Drazepinone (±)-**1**. Yellow oil; [α]_D_^25^ 0 (c 1.00, MeOH); UV (MeOH), λ_max_ (log *ε*) 206 (9.6), 247 (10.8) nm; ^1^H and ^13^C NMR data (see Table 1); HRESIMS *m*/*z* 294.1464 [M + H]+ (calcd for C_19_H_20_NO_2_, 294.1489), 316.1282 [M + Na]+ (calcd for C_19_H_19_NO_2_Na, 316.1300).

Compound (+)-**1**. [α]_365_ 130.4, [α]_436_ 92.2, [α]_546_ 54.6, [α]_589_ 36.3, [α]_633_ 21.8 (c 1.00, MeOH); ECD (0.12 mM, MeOH), λ_max_ (Δ*ε*) 213 (4.28), 225 (−2.57), 244 (10.3) nm.

Compound (−)-**1.** [α]_365_ −132.2, [α]_436_ −93.7, [α]_546_ −55.6, [α]_589_ −37.1, [α]_633_ −21.4 (c 1.00, MeOH); ECD (0.12 mM, MeOH), λ_max_ (Δ*ε*) 212 (−4.28), 226 (3.05), 244 (−11.4) nm.

### 3.3. Computational Section

Firstly, conformational searches of the molecules of the proposed structure of drazepinone and the revised structures of (2*R*,3*R*,4*S*)-**1** and (2*S*,3*S*,4*R*)-**1** were carried out using the MMFF94S force field by the ComputeVOA software, resulting in 4 stable conformers for drazepinone, 2 stable conformers for (2*R*,3*R*,4*S*)-**1**, and 2 stable conformers for (2*S*,3*S*,4*R*)-**1**, with relative energy within a 10.0 kcal/mol energy window, respectively. Secondly, the above minimum geometries were optimized at the gas-phase B3LYP/6-311+G(d) level using the Gaussian 09 package [17]. Finally, B3LYP theory at the basis sets of 6-311+G(d,p) in the gas phase was applied for ^13^C NMR calculations for both drazepinone and (2*R*,3*R*,4*S*)-**1**. Time-dependent density functional theory (TD-DFT) at the gas-phase B3LYP/6-311++G(2d,p) level was used for ECD calculations with a total of 60 excited states for (2*R*,3*R*,4*S*)-**1** and (2*S*,3*S*,4*R*)-**1**. A standard deviation of 0.20 eV was applied to ECD simulations for these calculations. VCD calculations for (2*R*,3*R*,4*S*)-**1** and (2*S*,3*S*,4*R*)-**1** were carried out at the B3LYP/6-311+G(d) level in gas phase. ORD calculations for (2*R*,3*R*,4*S*)-**1** and (2*S*,3*S*,4*R*)-**1** were performed at the B3LYP/6-311+G(d,p) level (Tables S2–S4). Boltzmann statistics were used for simulations of the ECD, VCD and ^13^C NMR of these molecules using SpecDis 1.64 [18].

### 3.4. Enzyme Inhibitory Activity Assay

PTP inhibitory activity of (+)-**1** and (−)-**1** against PTP1B, TCPTP, SHP1, and CD45 was tested in the same way as that described in the literature [19]. Na_3_VO_4_ was used as the positive control.

### 3.5. Molecular Docking

The complex crystal structure of PTP1B-inhibitor (PDB: 5T19) [20] or TCPTP-inhibitor (PDB: 2FJM) [21] was used as the starting model for molecular docking employing Discovery Studio 2017 software. Before docking calculations, conformational searches of compounds (+)-**1** and (−)-**1** were performed with GMMX conformer calculation (force field: MMFF94; energy window: 5.0 kcal/mol) was performed in GaussView 6.0. Then, the top 2 conformations with the lowest energy were optimized at the B3LYP/6-31G(d) basis set using density functional theory (DFT) in Gaussian 16. To simulate real conditions, the solvent effects of water were studied using the solvation model based on density (SMD). For the protein, protein preparation processes were carried out, such as removing water molecules, adding hydrogen atoms, and supplementing amino acid residues. The flexible docking protocol, which allows for some receptor flexibility during docking of flexible ligands [22], was used in this study employing CHARMm in Discovery Studio 2017 software. The receptor binding sites were determined from the location of ligand in complex PDB: 5T19 or 2FJM.

## 4. Conclusions

In conclusion, based on combinational analysis of 2D NMR data, ^13^C NMR chemical shift calculation, and computational studies of ECD, VCD, and ORD data, the structure of drazepinone was revised. Furthermore, experimental and molecular docking of the inhibitory effect between the revised (+)-**1** and (−)-**1** against PTPs were investigated. The complexity of intriguing structures and the significance of bioactivity for (+)-**1** and (−)-**1** may encourage further investigations on the chemistry and activity of this cluster of metabolites.

## Figures and Tables

**Table 1:** ^1^H (600 MHz) and ^13^C (150 MHz) NMR Data of (±)-**1** in CDCl_3_ and the corresponding NMR Data of drazepinone from [2].

No.	(±)-1 Measured in CDCl_3_		Drazepinone Measured in CDCl_3_	
	*δ*_C_ ^b^	*δ*_H_ ^d^	*δ*_C_ ^c^	*δ*_H_ ^d^
2	103.9	-	104.1	-
3	56.8	3.26 brs	56.7	3.26 brd (1.3)
4	49.1	2.89 brq (7.2)	49.1	2.90 brq (7.2)
5	150.5	-	150.5	-
6	126.3	5.38 s	126.3	5.38 brs
7	164.8 ^a^	-	164.9	-
8	113.5 ^a^	-	113.5	-
9	133.6	7.43 s	133.8	7.44 s
10	-	11.2 brs	-	12.4 brs
11	162.6 ^a^	-	162.8	-
12	111.4	-	111.2	-
13	26.3	1.65 s	26.3	1.65 s
14	20.3	1.29 d (7.2)	20.3	1.29 d (7.2)
15	14.8	1.72 s	14.8	1.72 brs
16	133.4	-	133.6	-
17	127.6	7.51 d (7.8)	127.6	7.51 d (7.6)
18	128.5	7.38 dd (7.8,7.8)	128.5	7.38 dd (7.6,7.6)
19	127.3	7.30 dd (7.8,7.8)	127.2	7.30 brs
20	128.5	7.38 dd (7.8,7.8)	128.5	7.38 dd (7.6,7.6)
21	127.6	7.51 d (7.8)	127.6	7.51 d (7.6)

^a^ Chemical shifts were detected in the HMBC spectra; ^b,c,d^ Chemical shifts were recorded at 150, 100, and 600 Hz, respectively.

**Table 2:** PTPs inhibitory activities of (+)-**1** and (−)-**1**.

Compounds	IC_50_ (μg/mL)			
	PTP1B	TCPTP	SHP2	CD45
(+)-**1**	>25.0	>25.0	>25.0	>25.0
(−)-**1**	1.56	12.5	>25.0	>25.0
Na_3_VO_4_	1.56	3.13	6.25	6.25

## Data Availability

Data are contained within the article or Supplementary Material.

## Supplementary Materials

The following are available online at https://www.mdpi.com/article/10.3390/md19120714/s1. Figures S1−S14, Table S1: 1D and 2D NMR, and HRESIMS spectra of compounds (±)-**1**; Figure S15: Calculated ECD spectrum of drazepinone; Tables S2–S4: The coordinate for the lowest-energy conformer of drazepinone and (±)-**1** in the calculations, respectively.
