# Supplementary material for: Structure Revision and Protein Tyrosine Phosphatase Inhibitory Activity of Drazepinone
Source: Mar Drugs. 2021 Dec 20;19(12):714. doi: 10.3390/md19120714 (PMC8708580; doi:10.3390/md19120714)
Supplement: Supplementary file 1 [file marinedrugs-19-00714-s001.zip › marinedrugs-1522604-supplementary.pdf]

Structure Revision and Protein Tyrosine Phosphatase Inhibitory Activity of Drazepinone

Fei Cao [1,*], Li Pan [2], Wen-Bin Gao [3], Yun-Feng Liu [1], Cai-Juan Zheng [4,*] and Ya-Hui Zhang [1,5,*]

[1] Key Laboratory of Pharmaceutical Quality Control of Hebei Province, Key Laboratory of Medicinal Chemistry and Molecular Diagnostics of Education Ministry of China, College of Pharmaceutical Sciences, Hebei University, Baoding 071002, China; liuyunfeng199011@163.com
[2] State Key Laboratory of NBC Protection for Civilian, Beijing 102205, China; bk6180b@163.com
[3] College of Life Sciences, Cangzhou Normal University, Cangzhou 061000, China; wenbinxing@yeah.net
[4] Key Laboratory of Tropical Medicinal Resource Chemistry of Ministry of Education, Hainan Normal University, Haikou 571158, China
[5] Key Laboratory of Marine Drugs, the Ministry of Education of China, School of Medicine and Pharmacy, Ocean University of China, Qingdao 266003, China
* Correspondence: caofei542927001@163.com (F.C.); 15689932652@163.com (Y.-H.Z.); caijuan2002@163.com (C.-J.Z.)

List of Supporting Information

Figure S1. ^{1}H NMR (600 MHz, DMSO-d_6) spectrum of compounds (±)-**1**

Figure S2. ^{1}H NMR (600 MHz, CD_3OD) spectrum of compounds (±)-**1**

Figure S3 ^{1}H NMR (600 MHz, $CDCl_3$) spectrum of compounds (±)-**1**, and the ^{1}H NMR of of drazepinone fom the literature

Figure S4. ^{13}C NMR (150 MHz, DMSO-d_6) spectrum of compounds (±)-**1**

Figure S5. ^{13}C NMR (150 MHz, CD_3OD) spectrum of compounds (±)-**1**

Figure S6. ^{13}C NMR (150 MHz, $CDCl_3$) spectrum of compounds (±)-**1**, and the ^{13}C NMR of drazepinone fom the literature

Figure S7. HSQC (DMSO-d_6) spectrum of compounds (±)-**1**

Figure S8. ^{1}H-^{1}H COSY (DMSO-d_6) spectrum of compounds (±)-**1**

Figure S9. HMBC (DMSO-d_6) spectrum of compounds (±)-**1**

Figure S10. HMBC ($CDCl_3$) spectrum of compounds (±)-**1**

Figure S11-12. Partial HMBC ($CDCl_3$) spectrum of compounds (±)-**1**

Figure S13. NOESY (DMSO-d_6) spectrum of compounds (±)-**1**

Figure S14. HRESIMS spectrum of compounds (±)-**1**

Figure S15. Calculated ECD spectrum of drazepinone

Table S1. ^{1}H (600 MHz) and ^{13}C (150 MHz) NMR Data of (±)-**1** in DMSO-d_6.and CD_3OD

Table S2. The coordinate for the lowest-energy conformer of drazepinone in ^{13}C NMR and ECD

calculations

Table S3. The coordinate for the lowest-energy conformer of (2*R*,3*R*,4*S*)-**1** in ^{13}C NMR, ECD, VCD, and ORD calculations

Table S4. The coordinate for the lowest-energy conformer of (2*S*,3*S*,4*R*)-**1** in ECD, VCD, and ORD calculations

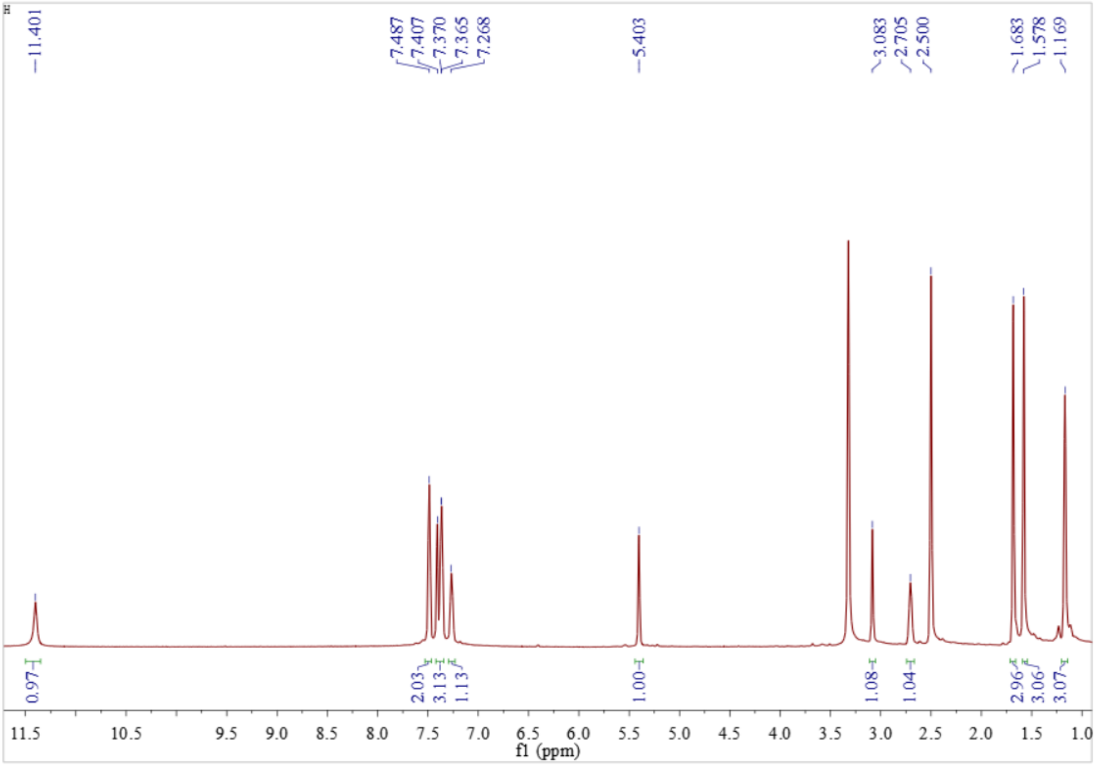

Figure S1. ^{1}H NMR (600 MHz, DMSO-d_6) spectrum of compounds (±)-**1**

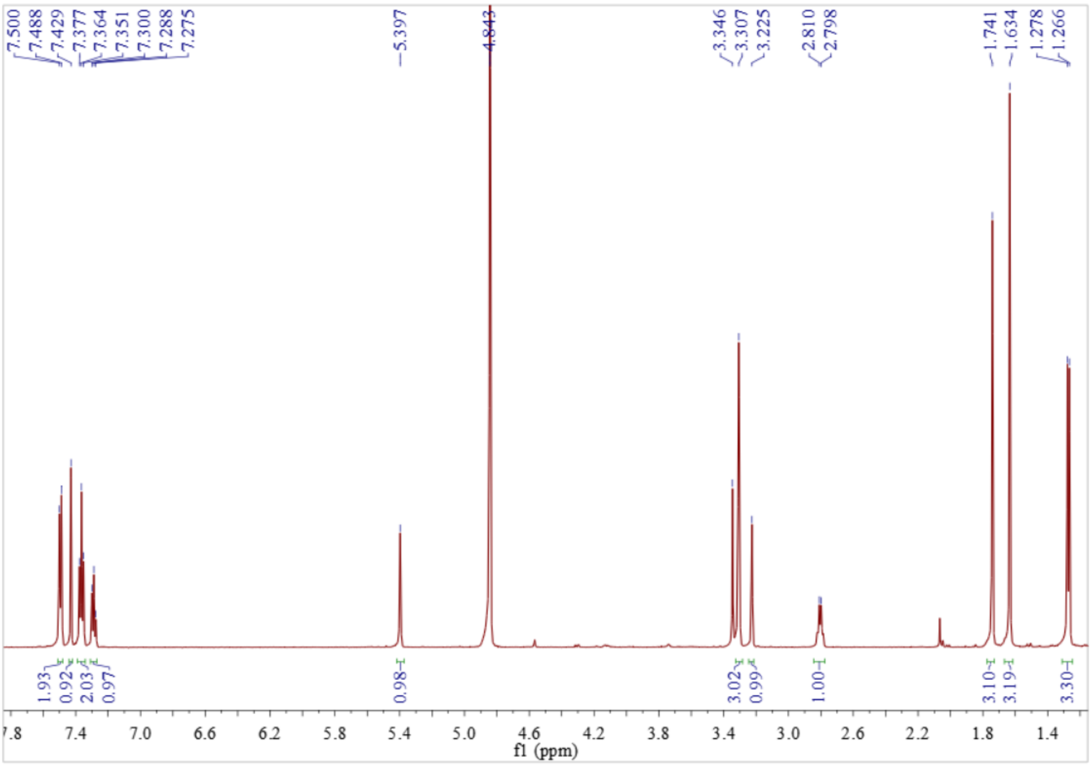

Figure S2. ^{1}H NMR (600 MHz, CD_3OD) spectrum of compounds (±)-**1**

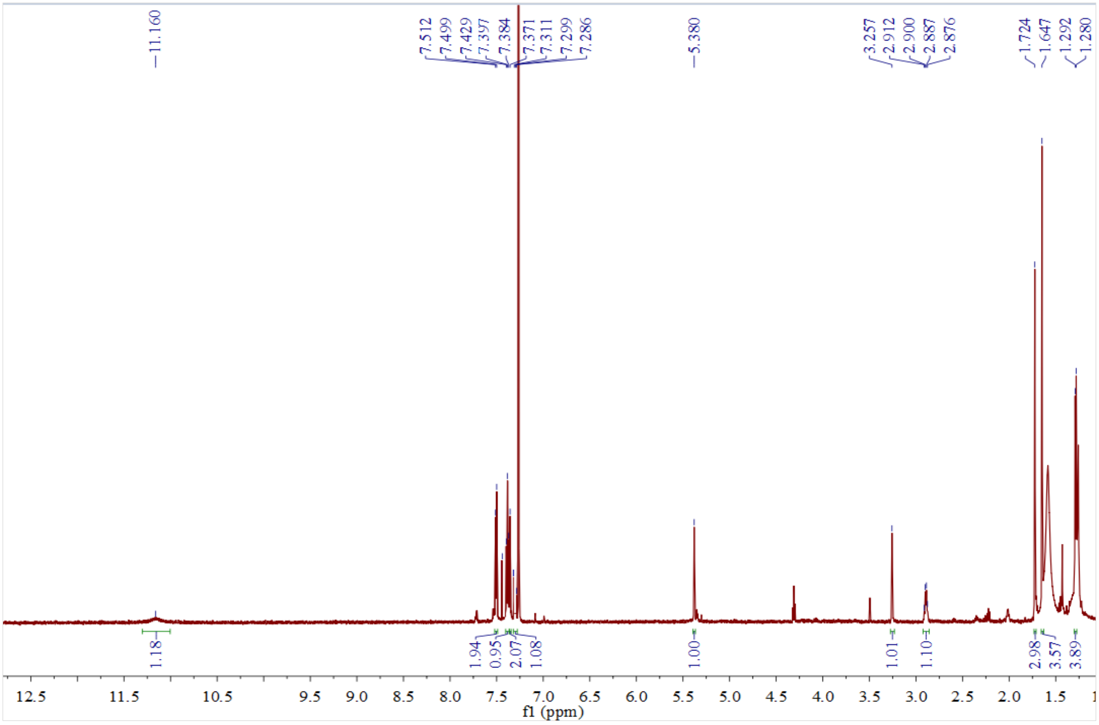

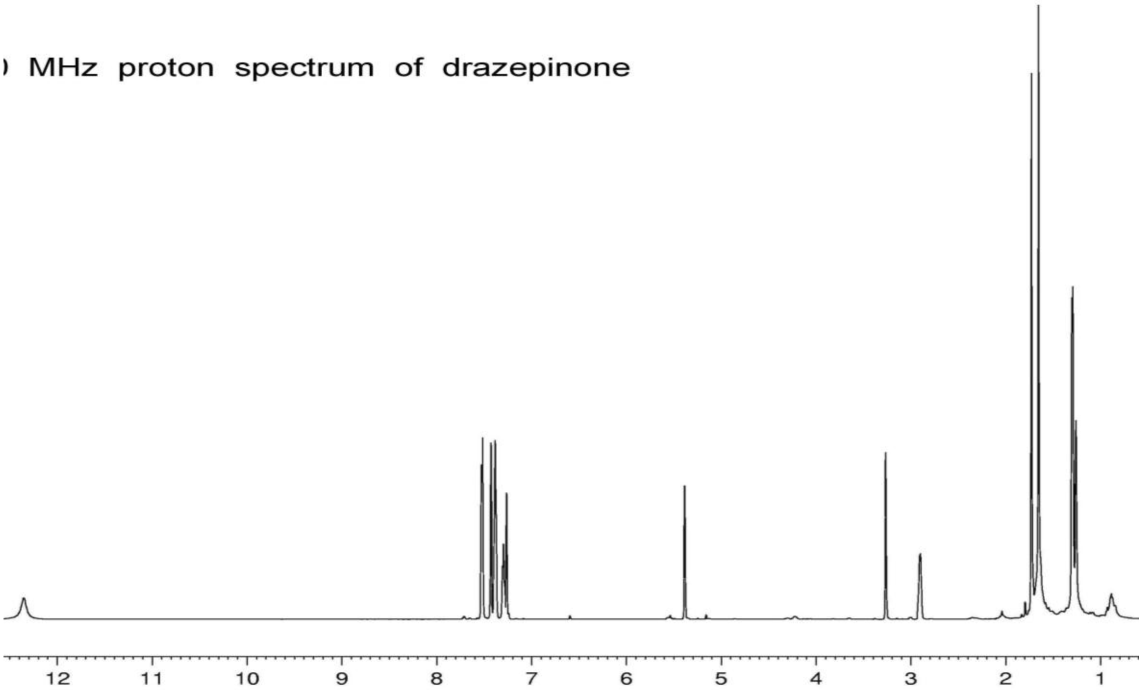

Figure S3 ^{1}H NMR (600 MHz, $CDCl_3$) spectrum of compounds (±)-**1**, and the ^{1}H NMR of of drazepinone fom the literature

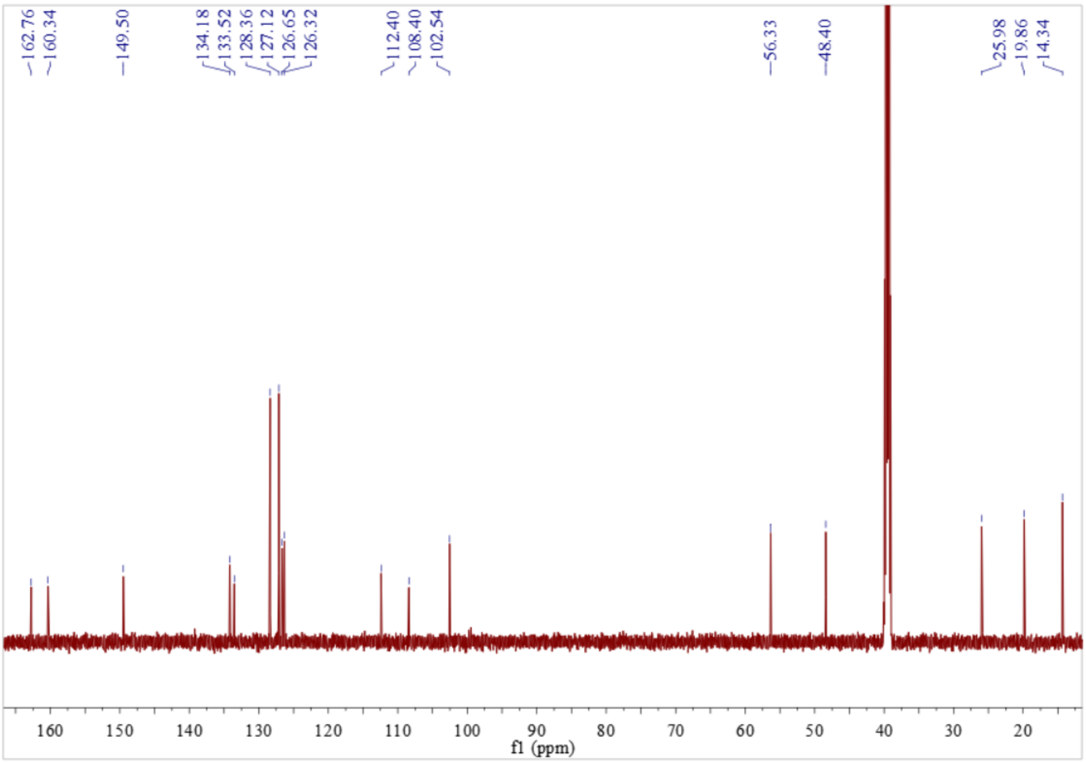

Figure S4. ^{13}C NMR (150 MHz, DMSO-d_6) spectrum of compounds (±)-**1**

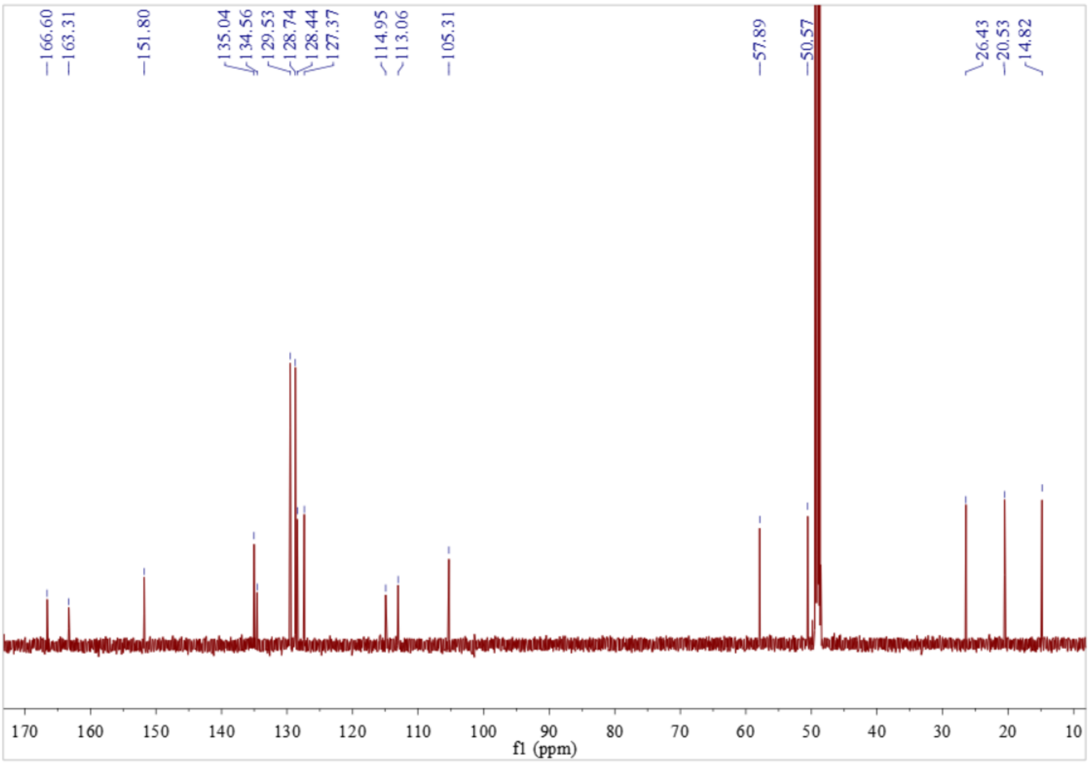

Figure S5. ^{13}C NMR (150 MHz, CD_3OD) spectrum of compounds (±)-**1**

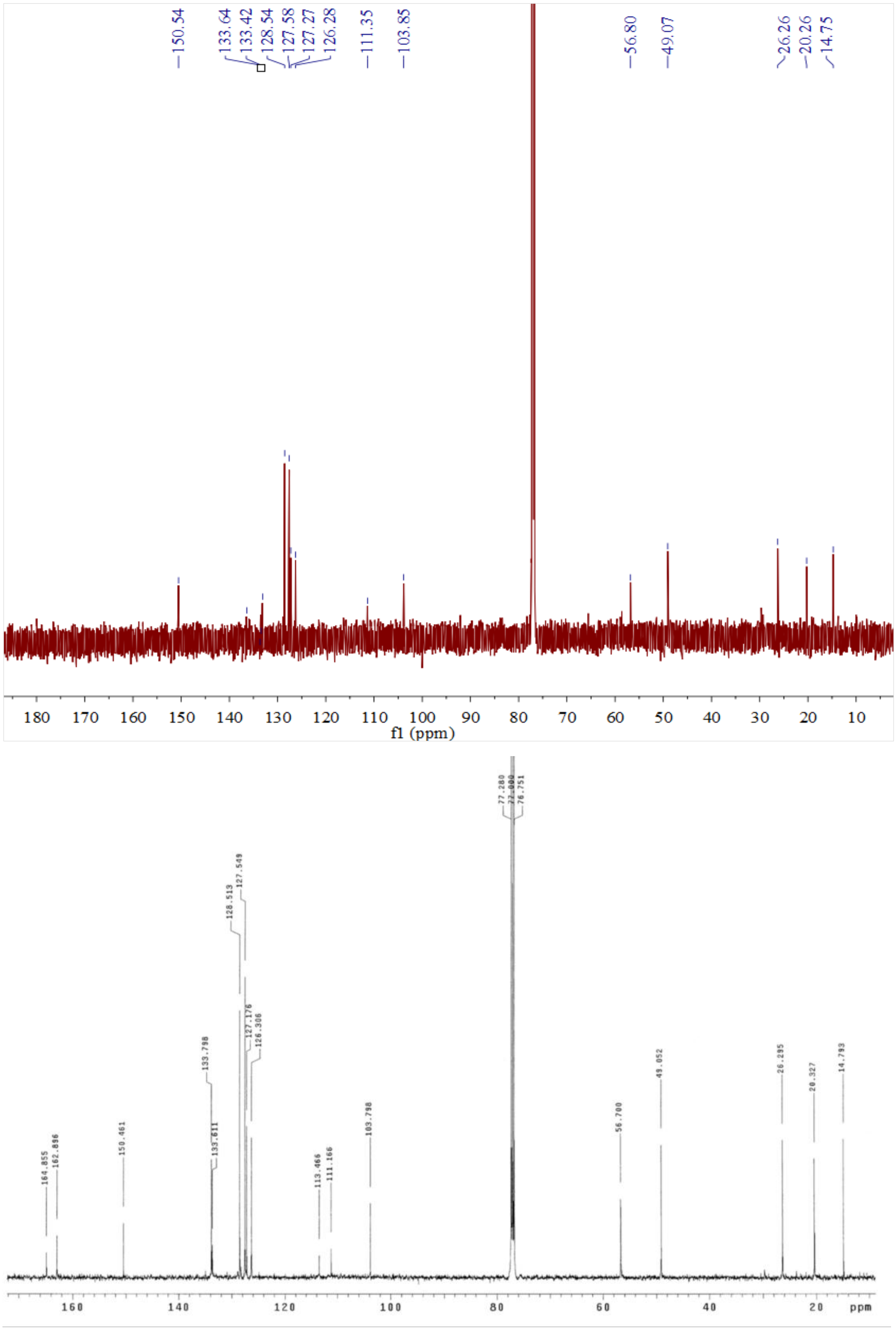

Figure S6. ^{13}C NMR (150 MHz, $CDCl_3$) spectrum of compounds (±)-**1**, and the ^{13}C NMR of of drazepinone fom the literature

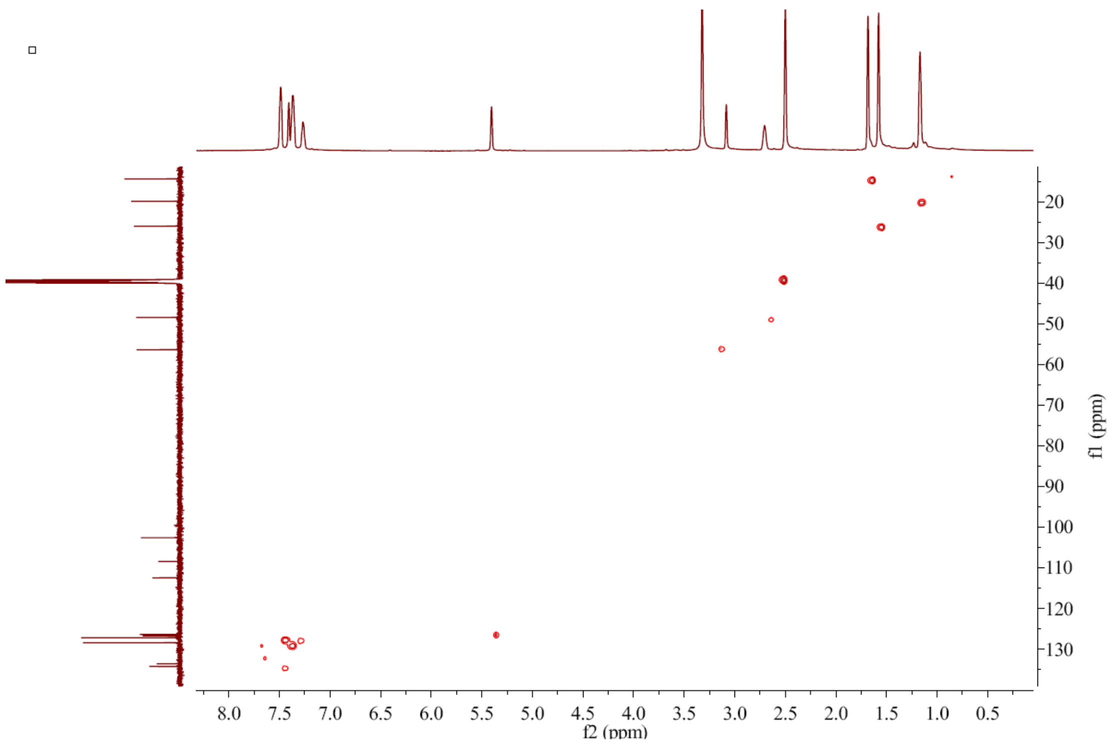

Figure S7. HSQC (DMSO-d_6) spectrum of compounds (±)-**1**

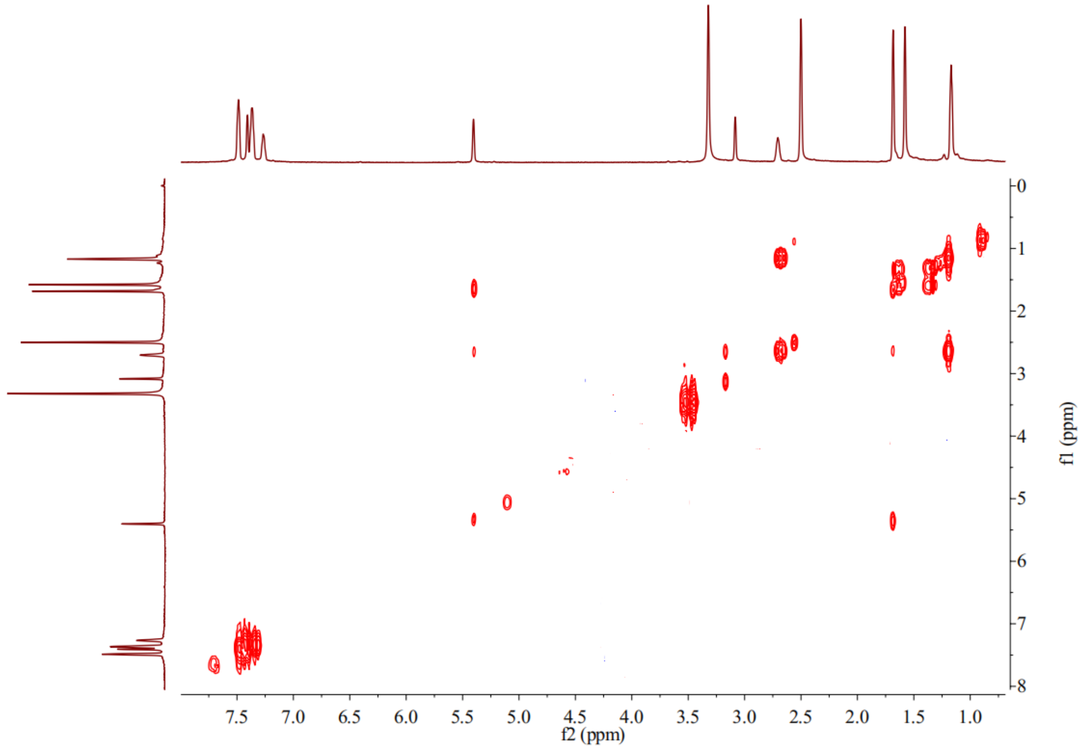

Figure S8. ^{1}H-^{1}H COSY (DMSO-d_6) spectrum of compounds (±)-**1**

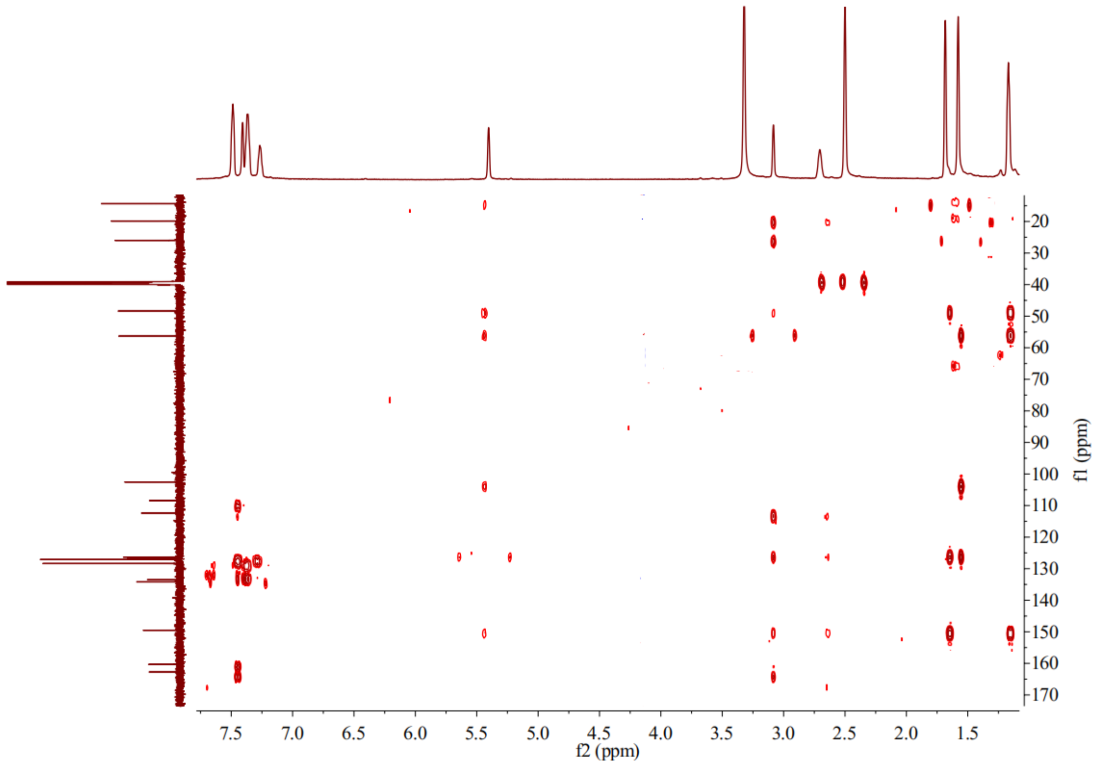

Figure S9. HMBC (DMSO-d_6) spectrum of compounds (±)-**1**

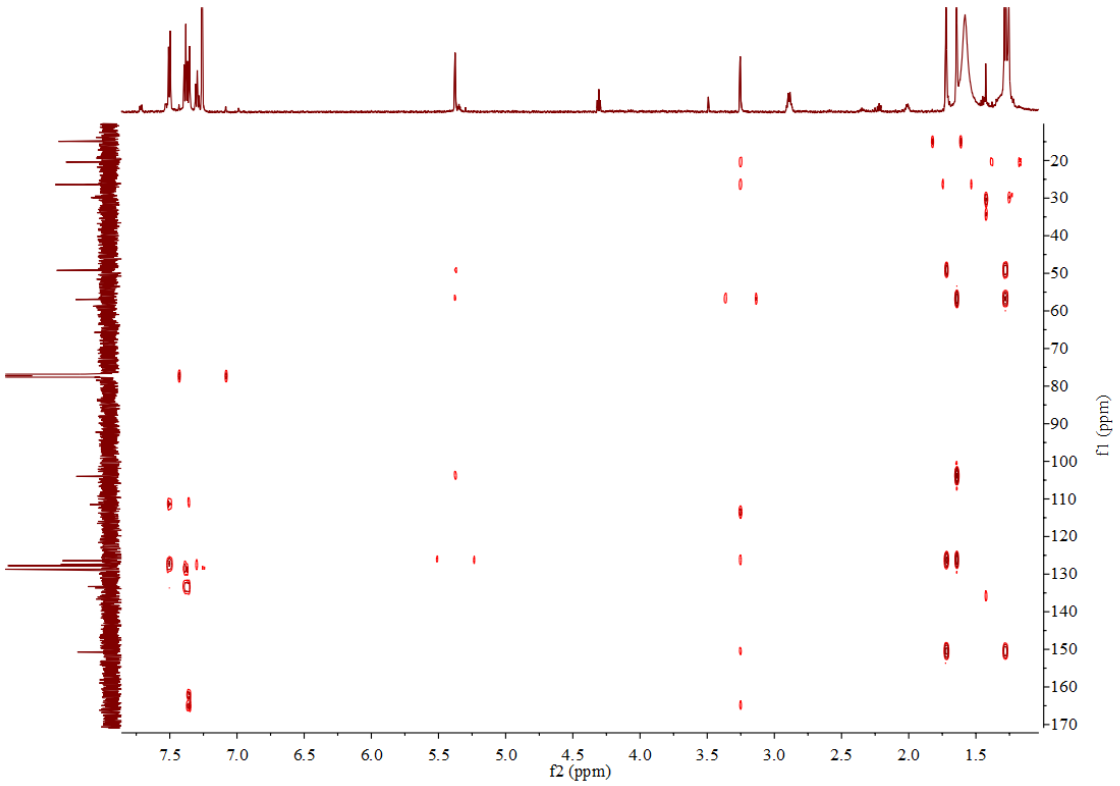

Figure S10. HMBC ($CDCl_3$) spectrum of compounds (±)-**1**

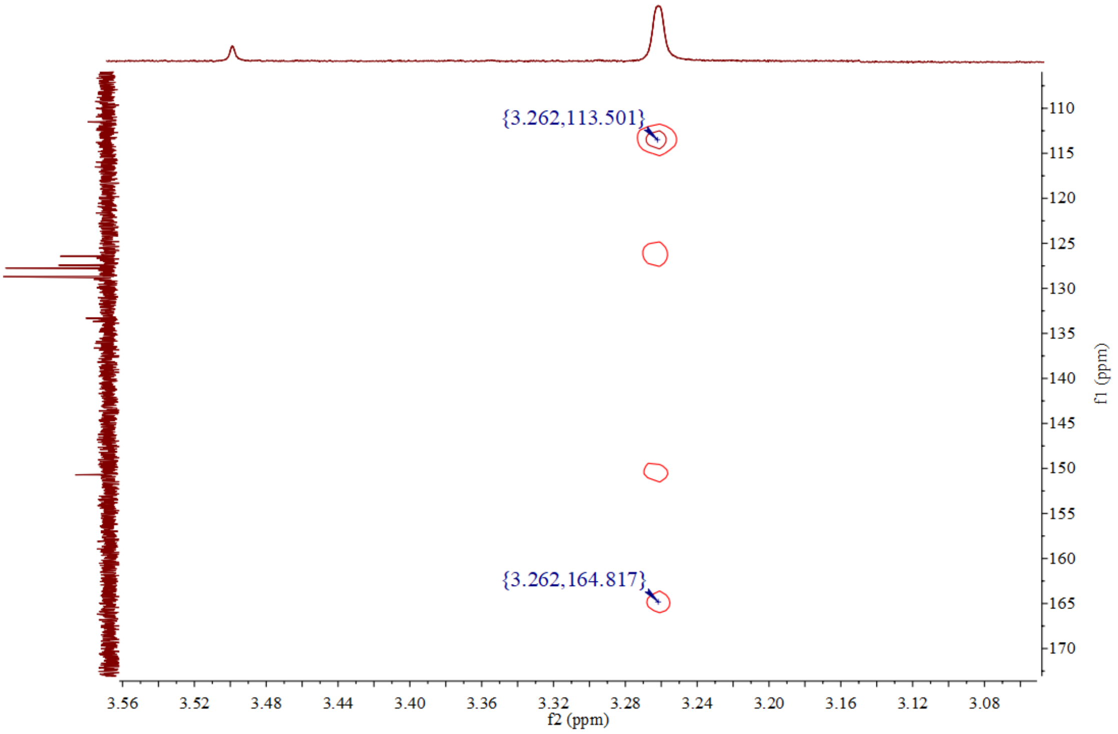

Figure S11. Partial HMBC ($CDCl_3$) spectrum of compounds (±)-**1**

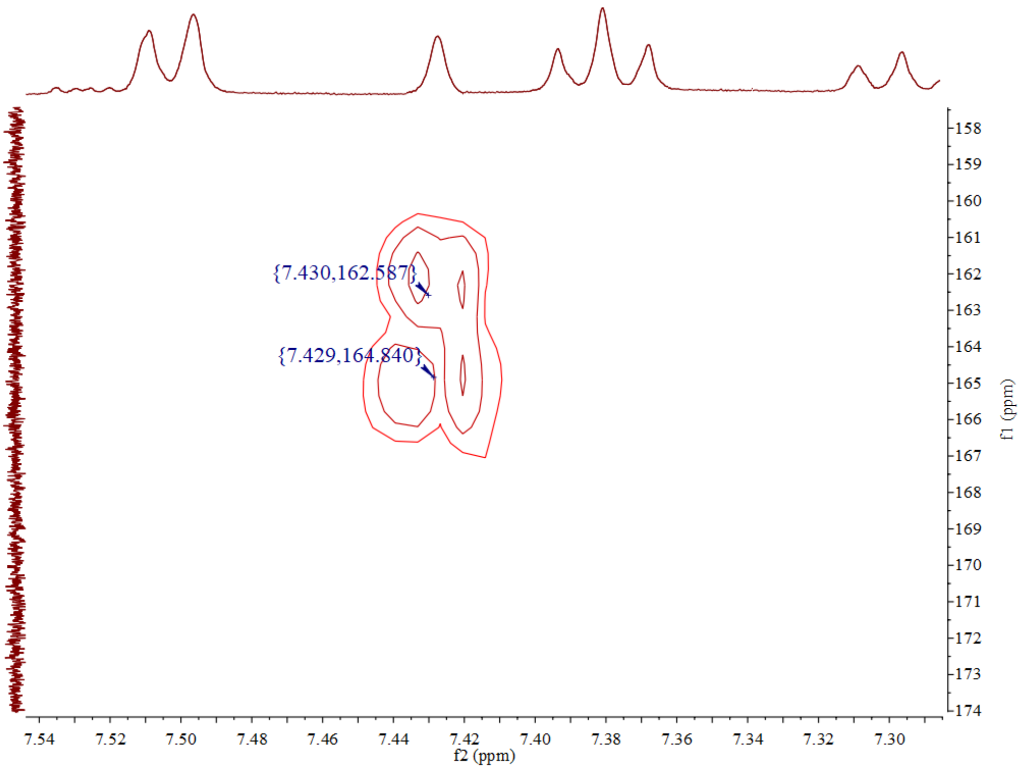

Figure S12. Partial HMBC ($CDCl_3$) spectrum of compounds (±)-**1**

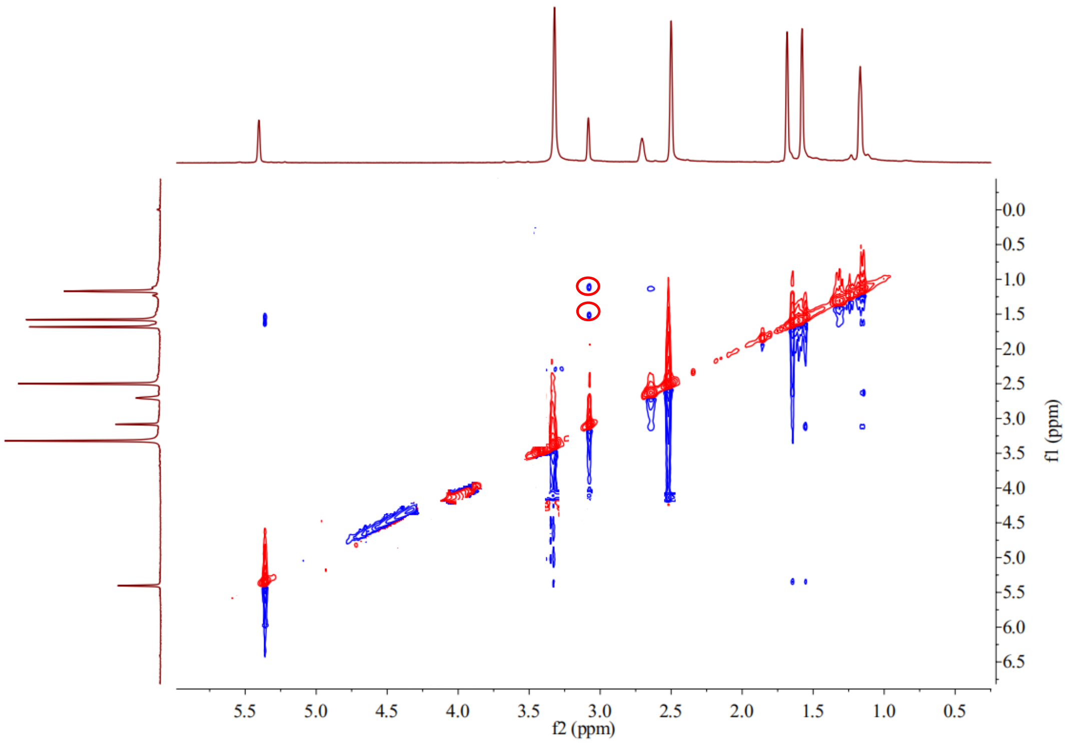

Figure S13. NOESY (DMSO-d_6) spectrum of compounds (±)-**1**

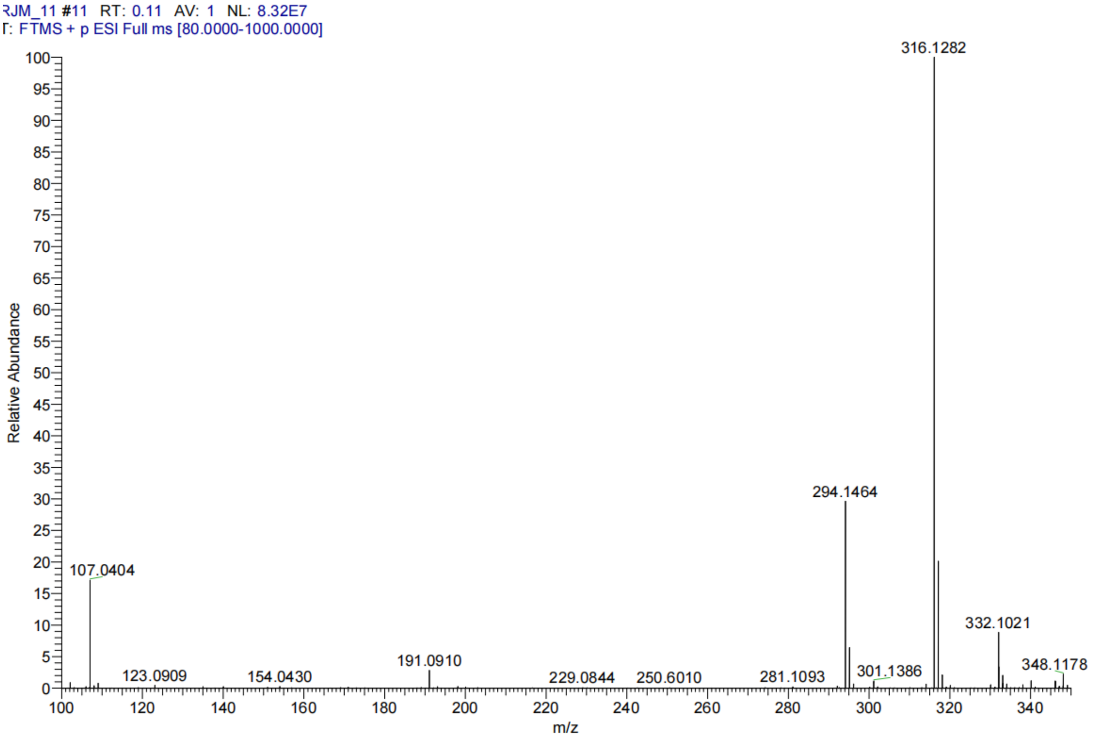

Figure S14. HRESIMS spectrum of compounds (±)-**1**

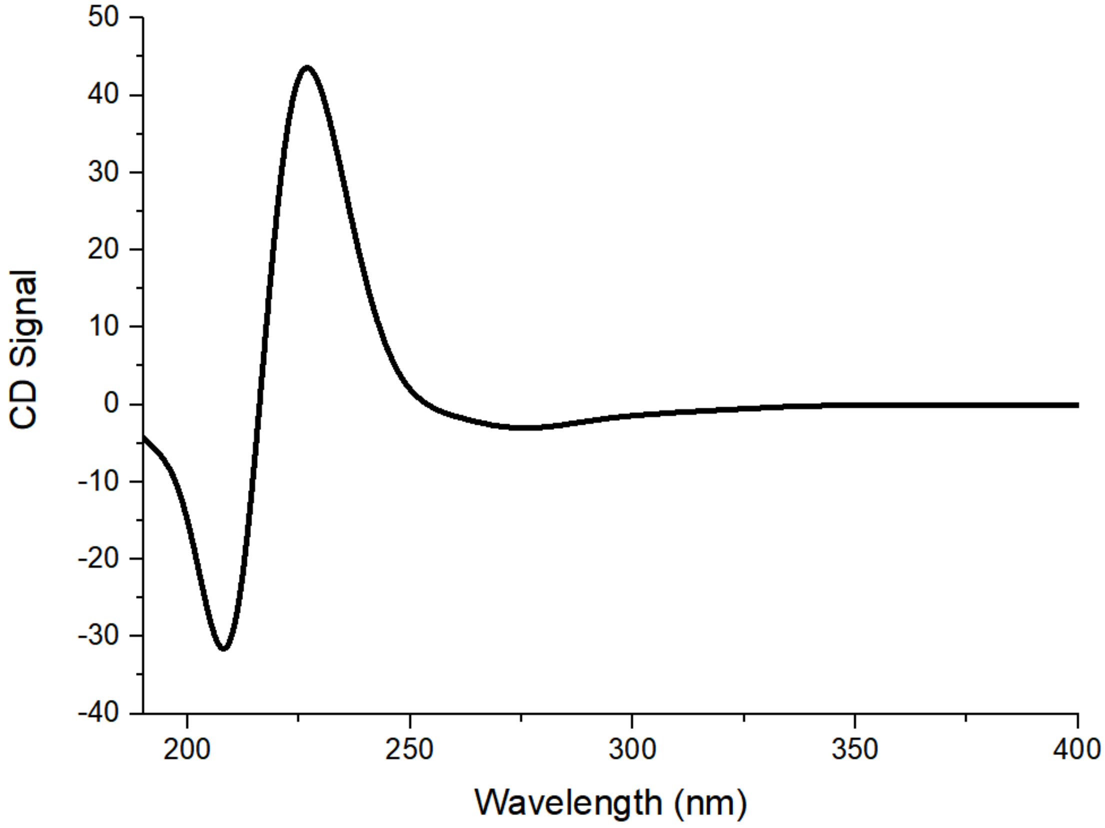

Figure S15. Calculated ECD spectrum of drazepinone

Table S1. ^{1}H (600 MHz) and ^{13}C (150 MHz) NMR Data of (±)-**1** in DMSO-d_6.and CD_3OD

No.	measured in DMSO-d_6		measured in CD_3OD	
	δ_C	δ_H	δ_C	δ_H
2	102.5	-	105.3	-
3	56.3	3.08 s	57.9	3.23 s
4	48.4	2.71 brs	50.6	2.80 q (7.2)
5	149.5	-	151.8	-
6	126.3	5.40 s	127.4	5.40 s
7	162.8	-	166.6	-
8	108.4	-	113.1	-
9	134.2	7.41 s	135.0	7.43 s
10	-	11.40 s	-	-
11	160.3	-	163.3	-
12	112.4	-	114.9	-
13	25.9	1.58 s	26.4	1.63 s
14	19.9	1.17 brs	20.5	1.27 d (7.2)
15	14.3	1.68 s	14.8	1.74 s
16	133.5	-	134.6	-
17	127.1	7.49 brs	128.7	7.49 d (7.2)
18	128.4	7.37 brs	129.5	7.36 d (7.8)
19	126.7	7.27 brs	128.4	7.29 dd (7.8, 7.2)
20	128.4	7.37 brs	129.5	7.36 d (7.8)
21	127.1	7.49 brs	128.7	7.49 d (7.2)

Table S2. The coordinate for the lowest-energy conformer of drazepinone in ^{13}C NMR and ECD calculations

	Coordinates (Angstroms) X	Y	Z
C	5.68256100	-0.38861100	-0.40148500
C	5.50375900	0.98217200	-0.10430600
C	4.24474800	1.47138900	0.15341000
C	3.10897100	0.62162400	0.12594100
C	3.29071500	-0.76967100	-0.17124300
C	4.60410400	-1.24125800	-0.43293000
C	1.79938200	1.11460600	0.38757300
C	0.72015200	0.26933000	0.34278900
C	0.93257100	-1.10284400	0.08336100
C	2.16516800	-1.63680900	-0.17748400
C	-0.75815900	0.49165500	0.59422400
C	-1.33017900	-0.97549700	0.51410700
O	-0.21655300	-1.83624300	0.16179500
C	-1.49791900	1.46287900	-0.36688600
C	-2.95632800	1.55003900	0.03679900
C	-3.88321400	0.59118200	-0.09133900
C	-3.55445300	-0.71779800	-0.73858500
N	-2.27311800	-1.20060600	-0.59002300
C	-0.88312300	2.86651200	-0.41067300
C	-5.32806300	0.78228000	0.28201700
O	-4.37527200	-1.31709200	-1.42011000
C	-1.87768200	-1.47295500	1.84917800
H	-0.90907900	0.86080500	1.61491300
H	6.67880700	-0.76818300	-0.60516500
H	6.36223200	1.64543500	-0.08116700
H	4.10399700	2.52436400	0.38122500
H	4.74631700	-2.29383700	-0.65975000
H	1.68231600	2.16416000	0.63446600
H	2.28459700	-2.69749800	-0.36956800
H	-1.43267800	1.03017900	-1.37046000
H	-3.27015800	2.50157500	0.46531300
H	-2.13837600	-2.09813200	-1.04048100
H	0.11746600	2.85724900	-0.84250800
H	-0.82350400	3.31473400	0.58723500
H	-1.49803800	3.52493500	-1.03017400
H	-5.96895300	0.65130800	-0.59283500
H	-5.65249500	0.03738600	1.01600900
H	-5.49906800	1.77599000	0.70050400
H	-2.75383300	-0.89067800	2.14267000
H	-2.16274500	-2.52389200	1.77050300
H	-1.11843500	-1.37433100	2.62912900

Table S3. The coordinate for the lowest-energy conformer of (2*R*,3*R*,4*S*)-**1** in ^{13}C NMR, ECD, VCD, and ORD calculations

	Coordinates (Angstroms)		
	X	Y	Z
C	1.52820200	0.86779200	-0.13198700
C	1.50621100	2.19688300	-0.46631400
N	0.36816000	2.94448200	-0.43354900
C	-0.91564300	2.47378200	-0.06771400
C	-0.89941100	1.08683400	0.27329700
C	0.24901400	0.33697000	0.24015400
C	2.78341000	0.08180300	-0.15400300
C	2.81704100	-1.22271700	-0.67020500
C	4.01077000	-1.93653200	-0.72258300
C	5.19664000	-1.36622400	-0.26096600
C	5.17604300	-0.07536700	0.26326500
C	3.98083800	0.63738400	0.32235400
C	-2.05749000	0.24036700	0.71439900
C	-1.36582900	-1.14294700	0.95400100
O	0.08020100	-0.94165500	0.62108000
C	-3.16374000	0.00571300	-0.36151000
C	-2.95042900	-1.43820500	-0.78302500
C	-1.99111000	-2.03924600	-0.07363800
O	-1.87749000	3.24011000	-0.07575600
C	-3.76033800	-2.05883800	-1.87911300
C	-1.39366400	-1.67240900	2.37899500
H	-2.50560900	0.63392800	1.63242300
C	-4.57831900	0.29508800	0.16199700
H	2.39926700	2.71591700	-0.79217500
H	0.39870900	3.91868400	-0.70258200
H	1.90322800	-1.67674600	-1.03357900
H	4.01480100	-2.94291500	-1.12950100
H	6.12562400	-1.92573000	-0.30150100
H	6.08882000	0.37419900	0.64155800
H	3.97197400	1.62788900	0.76665100
H	-2.99079300	0.66625100	-1.21939700
H	-1.67259100	-3.07012800	-0.19654600
H	-3.44313100	-3.08264600	-2.08814300
H	-4.82575600	-2.08196600	-1.62701900
H	-3.67248700	-1.47908900	-2.80501900
H	-0.84706000	-2.61594000	2.45288800
H	-0.93530900	-0.95773000	3.06746700
H	-2.42493700	-1.84782300	2.69565700
H	-5.33580200	0.12394800	-0.60777700
H	-4.65657100	1.33920400	0.47417600
H	-4.82435500	-0.33911100	1.02024300

Table S4. The coordinate for the lowest-energy conformer of (2*S*,3*S*,4*R*)-**1** in ECD, VCD, and ORD calculations

	Coordinates (Angstroms)		
	X	Y	Z
C	4.57832000	0.29508300	0.16200200
H	2.50560700	0.63392800	1.63242400
C	1.39366400	-1.67241300	2.37899200
C	3.76033500	-2.05883500	-1.87911700
O	1.87749100	3.24011100	-0.07575600
C	1.99110600	-2.03924400	-0.07364200
C	2.95042700	-1.43820400	-0.78302800
C	3.16374100	0.00571200	-0.36150700
O	-0.08020200	-0.94165300	0.62108100
C	1.36582700	-1.14294800	0.95399900
C	2.05749000	0.24036700	0.71440000
C	-3.98083900	0.63738600	0.32234900
C	-5.17604300	-0.07536600	0.26326000
C	-5.19663800	-1.36622500	-0.26096600
C	-4.01076700	-1.93653500	-0.72257800
C	-2.81703800	-1.22271800	-0.67019900
C	-2.78341000	0.08180400	-0.15400200
C	-0.24901400	0.33697100	0.24015400
C	0.89941100	1.08683400	0.27329600
C	0.91564400	2.47378300	-0.06771400
N	-0.36815800	2.94448200	-0.43354800
C	-1.50621000	2.19688500	-0.46631300
C	-1.52820200	0.86779300	-0.13198700
H	5.33580400	0.12394300	-0.60777100
H	4.65657400	1.33919900	0.47418300
H	4.82435400	-0.33911800	1.02024800
H	0.93531000	-0.95773500	3.06746600
H	0.84706000	-2.61594500	2.45288500
H	2.42493700	-1.84782700	2.69565200
H	3.44312700	-3.08264300	-2.08814900
H	3.67248600	-1.47908500	-2.80502200
H	4.82575300	-2.08196500	-1.62702300
H	1.67258500	-3.07012500	-0.19655500
H	2.99079700	0.66625400	-1.21939300
H	-3.97197700	1.62789300	0.76664300
H	-6.08882200	0.37420100	0.64154900
H	-6.12562300	-1.92573100	-0.30150100
H	-4.01479600	-2.94291900	-1.12949300
H	-1.90322400	-1.67674800	-1.03356800
H	-0.39870900	3.91868500	-0.70258000
H	-2.39926600	2.71592100	-0.79217200
